# Impact of Web Blight on Photosynthetic Performance of an Elite Common Bean Line in the Western Amazon Region of Colombia

**DOI:** 10.3390/plants11233238

**Published:** 2022-11-25

**Authors:** Juan Carlos Suárez, José Iván Vanegas, José Alexander Anzola, Amara Tatiana Contreras, Milan O. Urban, Stephen E. Beebe, Idupulapati M. Rao

**Affiliations:** 1 Programa de Ingeniería Agroecológica, Facultad de Ingeniería, Universidad de la Amazonia, Florencia 180001, Colombia; 2 Centro de Investigaciones Amazónicas CIMAZ Macagual César Augusto Estrada González, Grupo de Investigaciones Agroecosistemas y Conservación en Bosques Amazónicos-GAIA, Universidad de la Amazonia, Florencia 180001, Colombia; 3 International Center for Tropical Agriculture (CIAT), Km 17 Recta Cali-Palmira, Cali 763537, Colombia

**Keywords:** chlorophyll fluorescence, energy use, photosynthetic response, *Thanatephorus cucumeris*, acidic soil, high temperature

## Abstract

Disease stress caused by plant pathogens impacts the functioning of the photosynthetic apparatus, and the symptoms caused by the degree of severity of the disease can generally be observed in different plant parts. The accurate assessment of plant symptoms can be used as a proxy indicator for managing disease incidence, estimating yield loss, and developing genotypes with disease resistance. The objective of this work was to determine the response of the photosynthetic apparatus to the increased disease severity caused by web blight *Thanatephorus cucumeris* (Frank) Donk on the common bean (*Phaseolus vulgaris* L.) leaves under acidic soil and the humid tropical conditions of the Colombian Amazon. Differences in chlorophyll fluorescence parameters, including F_v_/F_m_, Y(II), Y(NPQ), Y(NO), ETR, qP, and qN in leaves with different levels of severity of web blight in an elite line (BFS 10) of common bean were evaluated under field conditions. A significant effect of web blight on the photosynthetic apparatus was found. A reduction of up to 50% of energy use dedicated to the photosynthetic machinery was observed, even at the severity scale score of 2 (5% surface incidence). The results from this study indicate that the use of fluorescence imaging not only allows for the quantifying of the impact of web blight on photosynthetic performance, but also for detecting the incidence of disease earlier, before severe symptoms occur on the leaves.

## 1. Introduction

The common bean (*Phaseolus vulgaris* L.) is the most important food legume for human consumption [1]. It is cultivated mainly in the tropical and subtropical countries of Latin America and Africa [2,3]. Beans are characterized as an accessible and economical food with high nutritional quality characteristics (protein, carbohydrate, micronutrients such as iron (Fe) and zinc (Zn)) [2] that are fundamental for reducing malnutrition, mainly in developing countries [3]. Likewise, bean production is mainly carried out by smallholders under adverse agroecological conditions that favor numerous infectious diseases caused by fungi, viruses, bacteria, and nematodes, resulting in yield losses of up to 100% [2,4,5,6,7].

Biotic stress caused by pathogens in plants inevitably induces various changes in their physiological functions, resulting in metabolic disorders due to infections or nutritional deficiencies [8]. Symptoms of biotic stress can generally be seen on leaves, stems, and roots, and lesions can also appear on seeds, leading to losses in yield and reduced grain quality [9]. Thus, an accurate assessment of plant symptoms can be used as a proxy indicator for monitoring diseases, estimating yield loss, and for developing genotypes with disease resistance [10]. Specifically, web blight (WB) caused by the fungus *Thanatephorus cucumeris* (Frank) Donk (anamorph: *Rhizoctonia solani* Kühn), is a great threat to achieving economic benefits from cultivating bean crops [11]. This is because the disease can develop in any developmental stage of the bean, mainly causing defoliation that can lead to the total loss of the crop in the short period of 1 to 3 weeks in areas with conditions favorable to the pathogen’s development [11,12]. The incidence and severity of WB is favored mainly by prolonged and sudden periods of high rainfall, under conditions of high humidity and temperatures above 23 °C, that are natural in the humid tropics [13].

Much of the research conducted to overcome this disease has focused on breeding to develop WB resistant genotypes [13]. However, the progress has been limited to developing some genotypes with a moderate level of resistance [14]. In addition, some strategies have been implemented for improving genetic resistance of bean lines to manage WB. These include the use of the biological control of fungal pathogens [15] and the inoculation with *Rhizobium* strains [16,17], as well as some soil and crop management strategies, such as minimum tillage, crop rotation, wider planting distances between plants, dry season planting, and the use of amendments and organic substrates [18,19,20,21]. There are also other strategies related to the use of bean lines with indeterminate growth habits because their plant architecture induces less favorable conditions for the development of WB [22].

In breeding programs, disease stress assessment is based on disease severity, which is defined as the area of plant tissue infected by disease-causing organisms, and it is usually expressed as a percentage of the total amount of plant tissue [11,23]. This is considered as an easy and rapid method for evaluating disease susceptibility [24]. However, in most cases, severity is measured based on the subjectivity of the evaluator, often lacking precision, efficiency/productivity, and traceability [25,26,27]. However, there are now other phenotyping methods that are based on the photosynthetic response to the degree of disease incidence [28,29], and these can allow for the identification of moderately disease-resistant bean lines using a disease susceptibility index (DSI) [30]. Thus, the degree of sensitivity of a given bean cultivar to the degree of disease severity is reflected at the physiological level, based on the pathogen-specific or non-specific mechanisms it uses to maintain its photosynthetic activity [31,32]. Therefore, this nondestructive phenotyping method is a way to reduce subjectivity and to obtain a clearer evaluation of disease severity using digital images to quantify the degree of incidence based on changes in leaf color [30,33]. 

Among plant responses to the environment, chlorophyll fluorescence (Chl*_a_*) is very sensitive to physiological changes in plants and is used to measure the physiological state of a plant under both biotic and abiotic stress conditions [24,34,35,36]. The use of the Chl*_a_* imaging method allows for the comparison of differences in photosynthetic efficiency between disease-affected and completely healthy areas of the leaves [37,38]. The Chl*_a_* imaging also allows for the tracking of the effects of foliar diseases that cause a decrease in the photosynthetic efficiency, thereby considered as a novel, non-destructive, and highly sensitive technique [37,39,40]. Chl*_a_* imaging is based on the amount of light energy absorbed by the chlorophyll molecules in photosystem II (PSII) and how this energy is distributed and utilized in different yield pathways, such as the effective photochemical quantum yield of the PSII photosystem Y(II), dissipation of regulated energy in the form of non-photochemical heat Y(NPQ), and dissipation of unregulated energy fluorescence Y(NO) [41]. If the quantum yield of one of the processes decreases, the quantum yield of another or both will increase [24,42]. One of the main strengths of Chl*_a_* fluorescence measurement is that it can detect many stress-induced changes before visible damage occurs [43,44]. The objective of this work was to determine the response of the photosynthetic apparatus to the increased severity caused by WB in leaves of an elite breeding line of common bean (*Phaseolus vulgaris* L.) grown under acidic soil, combined with the high humidity and temperature conditions of the Colombian Amazon. We tested the hypothesis that the adaptive response of the photosynthetic apparatus contributes to achieve tolerance to disease stress caused by WB in the common bean. Using Chl*_a_* imaging information, we determined the area visually affected by WB stress, as well as the total physiologically compromised area of the leaf, which allowed for a full evaluation of the impact of the degree of incidence of WB on photosynthetic performance.

## 2. Results

### 2.1. Severity of Web Blight on Common Bean Leaves

**Figure 1:**
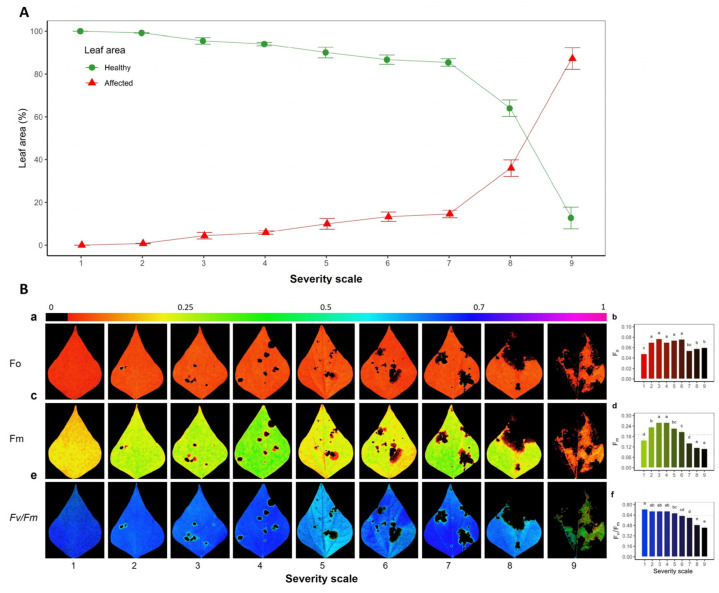
Severity of web blight on common bean leaves. (**A**) Area (%) of healthy and affected leaves according to the severity scale; the *x*-axis is related to the severity scale of 1 to 9, proposed by Schoonhoven and Pastor-Corrales (1987). (**B**) Chlorophyll fluorescence variables quantified during the dark adaptation phase: (**a**,**b**) initial fluorescence (F_0_); (**c**,**d**) maximum fluorescence images (F_m_); (**e**,**f**) PSII maximum quantum efficiency (F_v_/F_m_) images; (**b**,**d**,**f**) bar graph with mean values for each variable, respectively. The color gradient bar shown from violet to red or black indicates the maximum and minimum value of each variable, respectively, where healthy zones range from blue to violet, and disease affected zones range from green to black color. ^a,b,c,d,e^: Different letters within each severity scale in (**b**,**d**,**f**) subfigures indicate statistical differences according to Fisher’s LSD test of means (*p* < 0.05). Results include the mean ± SE of 27 leaves at each severity scale level of 1 to 9.

**Figure 2:**
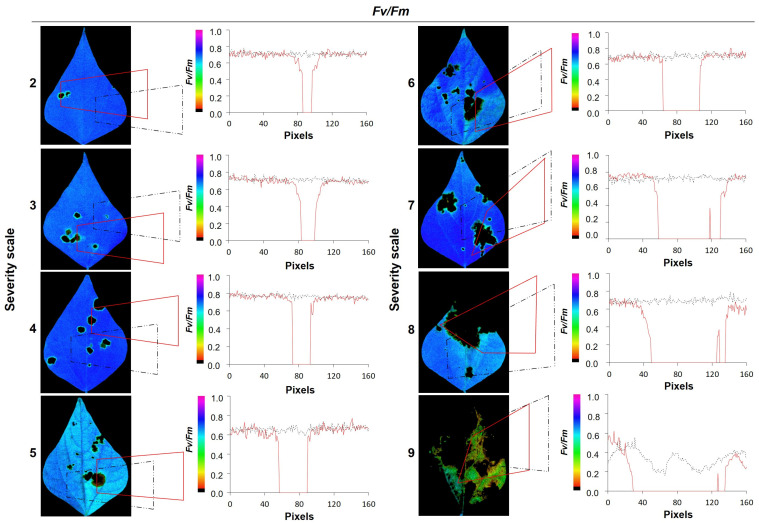
Measurement of F_v_/F_m_ along the transects for each severity scale of web blight on common bean leaves in healthy and affected areas. Subfigures 2 to 9 represent severity scale numbers. The right side of each image shows the changes in F_v_/F_m_ along the plotted zones; the solid red line shows “affected zones” and the dashed black line shows “apparently unaffected zones.” The color gradient from violet to red or black indicates the F_v_/F_m_ values, where healthy zones range from blue to violet, and affected zones range from green to black.

According to the severity scale of 1 (0% affected) to 9 (100% affected), there was an exponential growth behavior of the pathogen affected area in the leaf caused by WB stress (mm^2^ = 0.0796 exp ^(0.7759 × severity)^ (*p* < 0.001, Figure 1A), which resulted in a decrease in quantum efficiency, with an increase in the severity of stress level (F_v_/F_m_ < 0.78, Figure 1A). Visually, it was observed that a severity scale score of up to 7 affected the leaf area and was not greater than 15% of the total, which increased by two and six times at severity scores of 8 and 9, respectively (Figure 1A). At the physiological level, during the dark adaptation phase and using F_0_, the necrotic area caused by WB was more visible, and the color gradient went from yellow to red for F_m_ and from blue to green for F_v_/F_m_ (Figure 1B(a,b,c)). When the lesions caused by WB were analyzed by transects (Figure 2), it was found that at severity scale scores between 2 and 4, the values of F_v_/F_m_ were not affected. The values observed were above 0.8, while the values from a scale score of 5 and above were reduced. The length of the disease lesions described as pixels (diameter, red line) increased when the severity scale score increased; and the values observed ranged from 6% to 66.87% for scales 2 and 9, respectively.

### 2.2. Response of the Photosynthetic Apparatus to the Incidence of Web Blight

**Figure 3:**
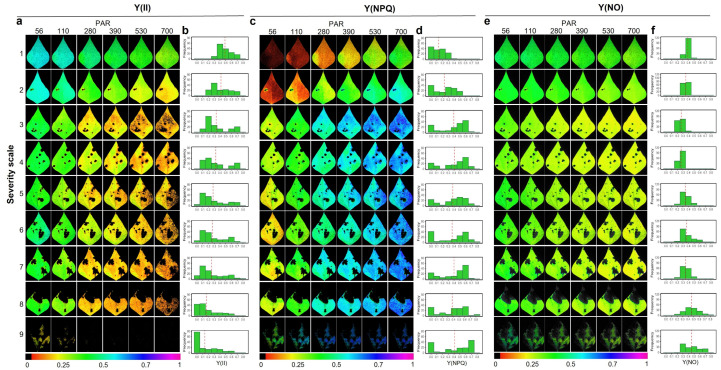
Energy allocation processes with an increase in photosynthetically active radiation (PAR) as a function of the increase in the degree of affected leaf area caused by Web blight and measured in different severity scales in common bean leaf blades. Results are shown as (**a**) images of the effective quantum yield in the PSII photosystem Y(II); (**b**) histograms of absolute frequencies of Y(II); (**c**). images of the quantum yield of the regulated energy dissipation Y(NPQ); (**d**) histograms of absolute frequencies of Y(NPQ), (**e**) images of the quantum yield of the unregulated energy dissipation Y(NO); and (**f**) histograms of absolute frequencies of Y(NO). Absolute frequency histograms correspond to 12 AOI (area of interest). The color gradient from violet to red or black indicates the maximum and minimum value of each variable, respectively, where healthy zones range from blue to violet, and affected zones range from green to black.

According to the analysis of variance (Supplementary Table S1), an effect of the evaluated factors (severity level and photosynthetically active radiation (PAR)) and their interaction (*p* < 0.001) was found on each of the Chl_a_ fluorescence parameters analyzed (F_0_, F_m_, F_v_/F_m_, Y(II), Y(NPQ), Y(NO), ETR, qP and qN). The relationship found for effective quantum yield in the PSII photosystem (Y(II)) and PAR was inversely proportional, a situation that was more evident (Figure 3a, going from blue to yellow, orange, and black) and frequent (Figure 3b) with increasing WB severity level, whose average value (red dotted vertical line, Figure 3b) of Y(II) also decreased. Likewise, as the severity increased, the fraction of energy dissipated as heat (NPQ) increased (red dotted vertical line, Figure 3d) and showed a very specific behavior; as the severity level increased, two peaks of data frequency were generated (Figure 3d). One of these is due to the increase in the necrotic area in the leaf caused by WB. This increased the value to zero, and increased those areas of the leaf showing a change in physiological functioning, which increased the dissipation of energy in the form of heat (NPQ), a situation that generated a gradient from green to blue-violet color (lower tendency to higher NPQ value, Figure 3c). In the case of the quantum yield of the unregulated energy dissipation of photosystem II, Y(NO) at all severity scale levels was reduced as the PAR level increased, whose average color was green (Figure 3e) and the average value ranged from 0.34 to 0.51 (red dotted vertical line, Figure 3f).

**Figure 4:**
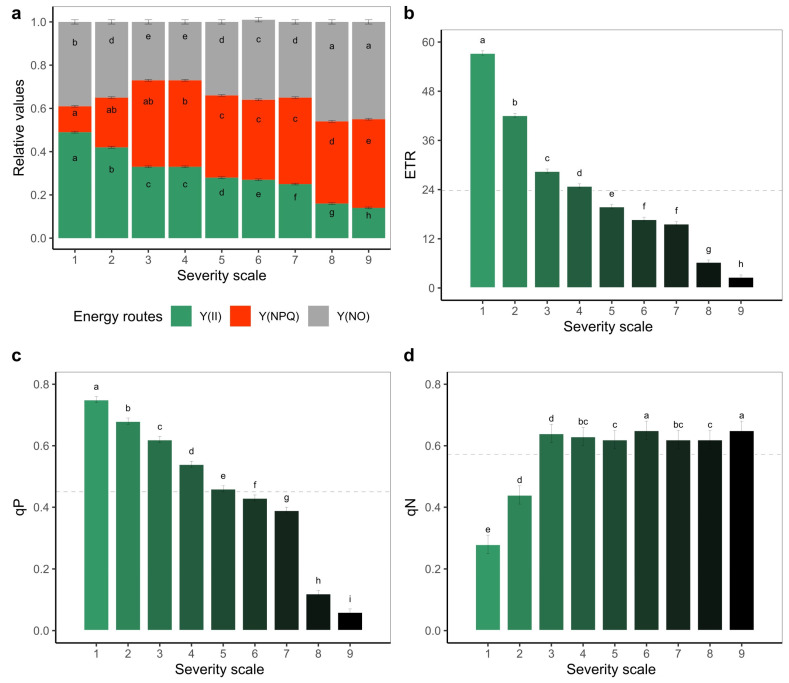
Chlorophyll fluorescence (Chl*_a_*) parameters as a function of an increasing degree of web blight impairment in common bean leaf laminae. Results are shown as (**a**) differences between severity scales in energy allocation ratios; each bar represents unity and they are divided into three complementary PSII performance terms: effective PSII quantum yield Y(II); regulated energy dissipation quantum yield Y(NPQ), and unregulated energy dissipation quantum yield Y(NO); (**b**) apparent electron transport rate (ETR); (**c**) photochemical quenching coefficient (qP); and (**d**) non-photochemical quenching coefficient (qN). ^a,b,c,d,e,f,g,h,i^: Different letters within each severity scale indicate statistical differences according to the Fisher LSD means test (*p* < 0.05). The black dotted line in each panel represents the average value of the variable. Results include mean ± SE (n = 1296).

Regarding the fractionation of energy use independent of disease severity score, on an average about 30% and 34% of its total value was assigned to photosynthetic processes Y(II) and heat dissipation Y(NPQ), respectively, and the remaining 36% was assigned to unregulated processes Y(NO) (Figure 4a). Specifically, with an increasing WB severity score, Y(II) decreased and Y(NPQ) increased. Likewise, the pathogen caused reductions of the electron transport rate (ETR), as it decreased from 50.3% to 95.3% at severity scores 1 to 9, respectively (Figure 4b). The same trend was observed with the photochemical quenching coefficient (qP), which reached values below 0.5 from a severity score of 5 onwards (Figure 4c). In the case of the non-photochemical cooling coefficient (qN), it was higher than 0.6 with a severity score of 3, at which time the presence of necrotic tissue became evident (Figure 4d).

**Figure 5:**
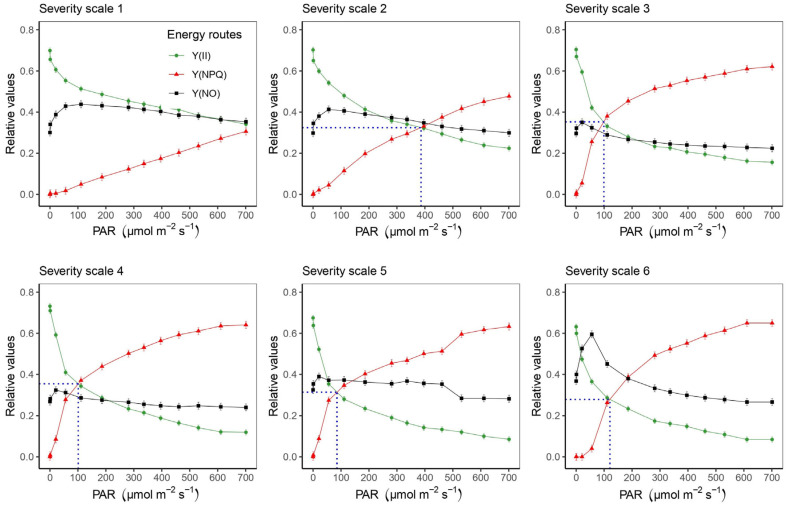

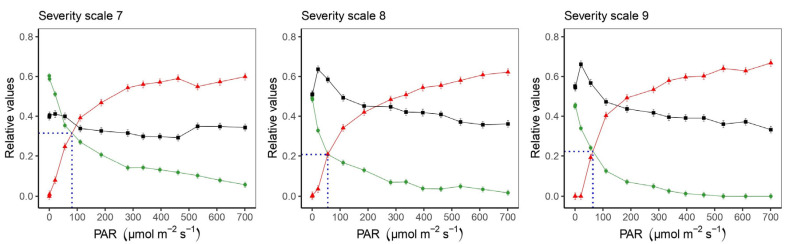
Effective quantum yield in photosystem II [Y(II)]; quantum yield of regulated energy dissipation [Y(NPQ)] and quantum yield of unregulated energy dissipation [Y(NO)]; fluorescence parameters that show the physiological deterioration caused by web blight in bean leaf blades as its degree of severity of stress increases; deterioration that is evidenced in a rapid light curve (RLC) at different photosynthetically active radiation (PAR) levels, when Y(II) is exceeded by Y(NPQ) and Y(NO). The blue dashed line shows the PAR level when Y(II) and Y(NPQ) intersect. The blue dotted line represents the crossover between the Y(II) curve and the Y(NPQ) curve. The sum of the energy allocation of the three routes = 1 at each PAR level. Results include mean ± SE (n = 144).

**Figure 6:**
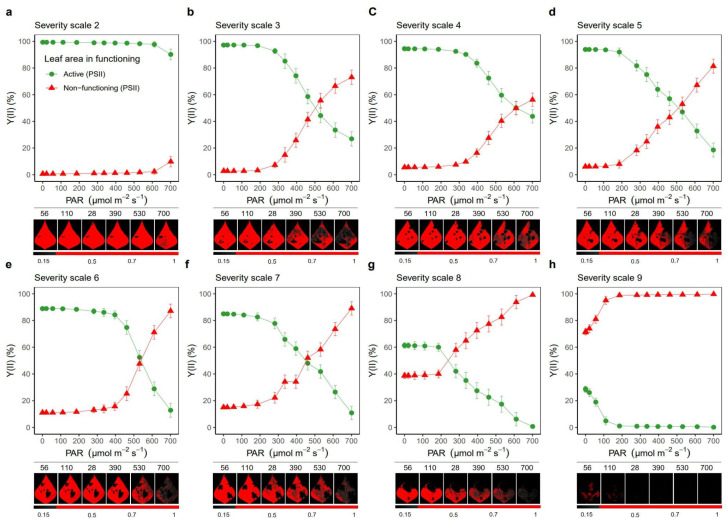
Effect of the pathogen on leaf area and photosynthetic apparatus functioning in relation to the level of radiation at each scale (subfigures (**a**–**h**)) of web blight severity. Green (active) and red (not active) color lines show the functioning of the photosynthetic apparatus in relation to the effective quantum yield in photosystem II [Y(II)]. The red color of the leaf indicates the functional area; the black color indicates the necrotic area caused by the pathogen. Results include mean ± SE (n = 144).

With regard to the effect of PAR level, it was found that between a severity scale score of 1 and 2, there was a 25% reduction in the functioning of the photosynthetic apparatus (Supplementary Figure S1). This is supported by the behavior of different parameters, such as Y(II) and ETR, which decreased with increasing PAR, a situation that also occurred with the fraction of energy devoted to photochemical processes (qP). For example, at a PAR of 700 µmol photons, m^−2^ s^−1^, which corresponds to the maximum photon flux density (PFD) generated in this study, it was observed that Y(II) was reduced by 35.2% with a severity scale score of 2 compared to a score of 1, increasing up to a 50% reduction with a scale score of 3 (Supplementary Figure S1a). Similar behavior was observed for ETR, where at the maximum PAR level, there was a 34.3% and 54.3% reduction with severity scale scores of 2 and 3, respectively, compared to a score of 1 (Supplementary Figure S1b). It was found that from a PAR of 300 µmol photons m^−2^ s^−1^ at severity scale level 3, the ETR did not show a significant increase (Supplementary Figure S1b). In the case of qP, which averaged 0.45 with a severity scale score of 1, the ETR decreased significantly from a severity scale score of 2 (Supplementary Figure S1c). For Y(NPQ) and qN, there was a proportional increase in relation to an increase in PAR, with the values at severity scales of 1 and 2 being the lowest compared to those at the other levels. When analyzing the energy distribution as a function of PAR at each severity scale, it was found that there was no point of intersection of Y(II) and Y(NPQ) at severity scale 1, even at the maximum PAR level (700 µmol photons m^−2^ s^−1^) (Figure 5). However, as the severity level increases, the intersection point between Y(II) and Y(NPQ) is reduced (area between the blue dotted lines) (Figure 5). It is also observed that from severity scale score of 4, the unregulated fraction (Y(NO)) exceeded the photosynthetic processes (Y(II)) (Figure 5). When we analyzed the area of leaf in operation for each severity scale, we found a significant effect of WB on the photosynthetic apparatus, since from severity scale score of 2 onwards, the use of energy devoted to the photosynthetic machinery was reduced by up to 50%, which was significantly reduced with the increasing value of the severity scale (Figure 6).

### 2.3. Correlation Coefficients between the Increase in Photosynthetically Active Radiation (PAR) and Physiological Variables at Different Severity Scales of Web Blight

**Figure 7:**
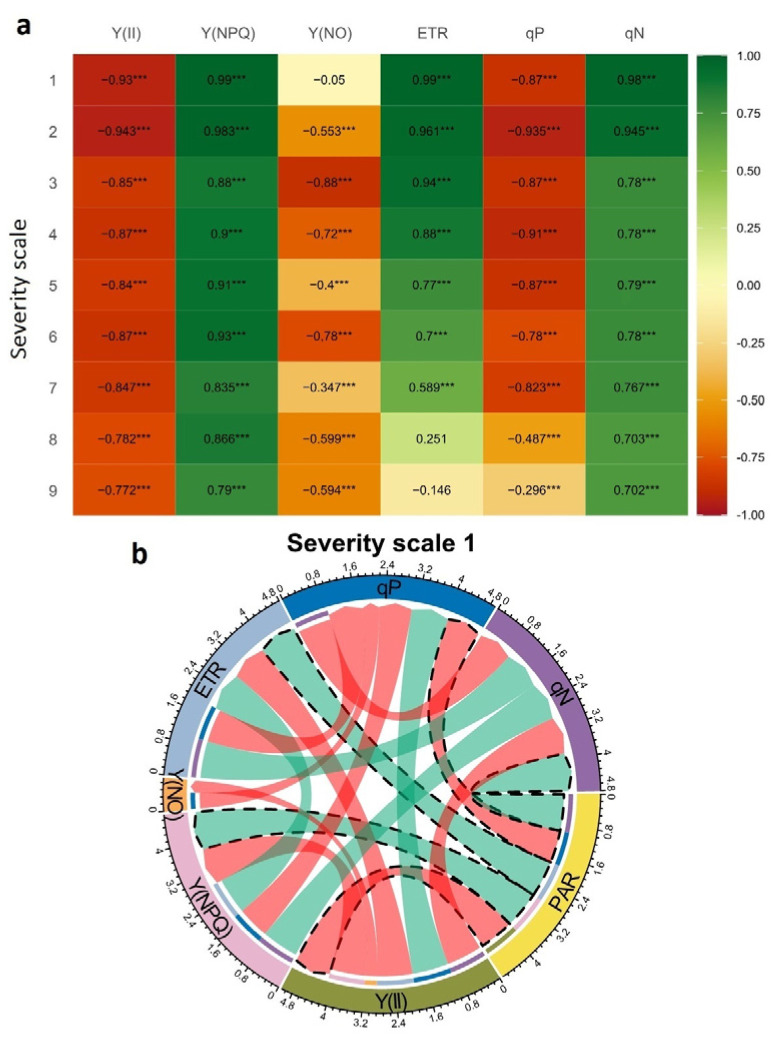

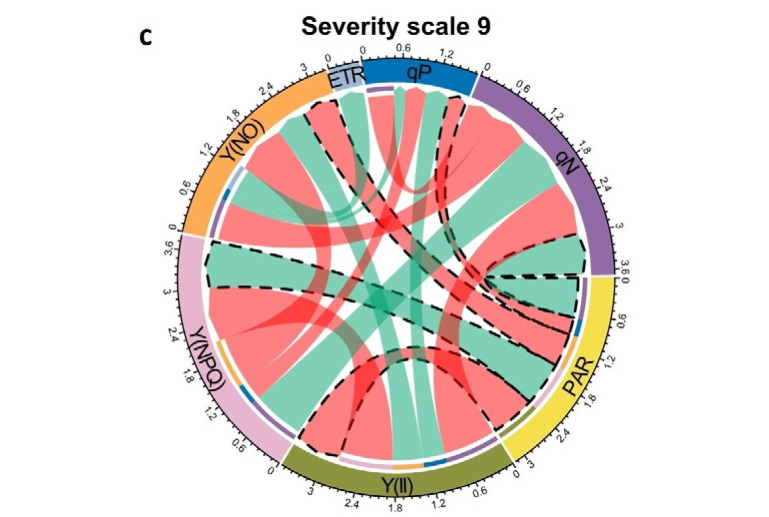
Correlation coefficients between chlorophyll fluorescence (Chl*_a_*) variables as photosynthetically active radiation (PAR) increases to show the disruption in the function of the photosynthetic apparatus with an increase in disease severity scales of web blight. Results are shown as: (**a**) the heat map of the Pearson correlation coefficients between a PAR increase and fluorescence variables for each severity scale. The color gradient from green to red indicates the magnitude of positive to negative correlation. *** represent significant at probability level of 0.001. The circles correspond to the chord diagram of the correlation coefficients between PAR increase and fluorescence variables; (**b**) severity scale 1, “no visible symptoms”; and (**c**) severity scale 9, “very severe disease symptoms”. Ribbons inside the circle correspond to significant correlations with a *p*-value < 0.05; green ribbons indicate positive coefficients, and red ribbons indicate negative coefficients.

When analyzing the relationship of increased PAR on the physiological variables, a high negative correlation (r ≥ −0.30, *p* < 0.05) was found with the variables (Y(II): effective quantum yield of PSII, and qP: photochemical quenching coefficient). A high positive correlation (r ≥ 0. 70, *p* < 0.05) was found with the variables (Y(NPQ): regulated energy dissipation quantum yield, and qN: non-photochemical cooling coefficient) for the analyzed factor of “severity scales” (Figure 7a). Although the increase in PAR showed a negative correlation with Y(II) and qP and a positive correlation with Y(NPQ) and qN at each severity scale, its correlation coefficient value decreased as the WB severity scale increased (Figure 7a). Similarly, the electron transport rate (ETR) correlated positively with increasing PAR, but its correlation value decreased with increasing severity to the point of being negative. As for the quantum yield of unregulated energy dissipation Y(NO), its correlation coefficient was found to be negative with increasing PAR and severity level (r ≥ −0.35, *p* < 0.05, Figure 7a), except for at severity scale 1, which presented no correlation with increasing PAR. When considering the minimum (Figure 7b) and maximum (Figure 7c) values of the severity scale, there were differences in the magnitude of the correlation between the variables. For example, at severity scale 1, PAR presented a positive correlation of 0.99 with ETR and Y(NPQ), while it was 0.98 for qN. However, for this same severity scale, there was a negative correlation between PAR with Y(II) and qP of −0.93 and −0.87, respectively. For example, Y(II) and qP were positively correlated (r = 0.98, *p* > 0.05) and were negatively correlated with qN, ETR, Y(NPQ), Y(NO), and PAR (Figure 7b). At the maximum scale of severity, the magnitude of correlation between variables is reduced, and the results for additional variables are also presented (Figure 7c). For example, PAR presented a negative correlation with Y(II), Y(NO), and qP with values of −0.77, −0.59, and −0.3, respectively, and a positive correlation with Y(NPQ) and qN, with values of 0.79 and 0.7, respectively. Among the other variables, Y(II) correlated positively with Y(NO) and qP and negatively with the other energy fractions Y(NPQ) and qN, with values of −0.91 and −0.92, respectively. 

## 3. Discussion

In general, we found that the classic severity scale proposed to identify the degree of infestation by a pathogen, particularly the one caused by WB, is very subjective. This is mainly because it is based on visual symptoms and not on the actual functioning of the photosynthetic apparatus of the leaf. Results from the present study show that with the severity scale score of 2, the area visually affected in the leaf does not exceed 5%, but the functional status of the photosynthetic apparatus is already compromised by more than 50%, which significantly affects the use of energy. Therefore, the use of chlorophyll fluorescence (Chl*_a_*) imaging as a tool allowed us to quantify the impact of the incidence of WB on photosynthetic efficiency (F_v_/F_m_), as well as the response of the functional status of the photosynthetic apparatus in relation to the increase in disease severity in the leaves. The specific changes in photosynthetic characteristics resulting from the increase in disease severity are discussed below.

### 3.1. Quantifying of Web Blight Incidence Using F_v_/F_m_ Chlorophyll Fluorescence Imaging

With the increase in disease severity, different combinations of changes in chlorophyll fluorescence parameters have been observed in the leaf. Changes in photosynthetic efficiency traits (increases and decreases in the different parameters) provide information about the plant’s ability to cope with the type of stress [45]. Our results indicate that the dark-adapted fluorescence images could be used for the presymptomatic detection of disease effects at the early stages of disease development. This is an important result, allowing for the observation of the different responses in chlorophyll fluorescence parameters as the severity intensifies with disease development. Specifically, we observed changes related to the increase in initial fluorescence (F_0_), reduction in maximum fluorescence (F_m_), and maximum efficiency of PSII photochemistry (F_v_/F_m_). We found that as the WB severity scale increased from level 2 to level 6, increases in F_0_ were present in the infected areas. This could indicate that the infection generated in the infected areas supports a breakdown of chlorophyll, thereby decreasing the reabsorption of red fluorescence light [46,47], triggering a loss of excitation energy, which is required during the electron transfer from pigments to the reaction centers [48]. Furthermore, for the higher disease severity scale scores of 7, 8, and 9, the decrease in values of F_0_ were more drastic, possibly due to the change in the rate of electron transport from PSII to the primary electron acceptors, accompanied by a more drastic reduction in the number and size of active reaction centers [49,50,51]. At the same time, a decrease in F_m_ was present in the affected areas, starting at a severity scale score of 5. This indicates that WB infestation generated strong alterations in the light-harvesting complexes of the PSII reaction centers [52,53,54]. Moreover, maximal quinone reduction may not have occurred because the reaction centers may not function properly due to an operationally inefficient energy capture process [55,56]. 

We found that in healthy areas (unaffected by WB), there were no variations in the ratio of F_v_/F_m_. Interestingly we found that in the case of the affected areas, between scale scores of 2 and 4, the efficiency of PSII was not compromised, as revealed by sustained values above 0.8 [27]. However, from scale 5 onwards, there was a reduction in F_v_/F_m_ values to 0.6 until reaching values of up to 0.4 with a scale score of 9, indicating the impact of WB in markedly reducing photosynthetic efficiency. Disease severity could induce physical damage to the structure and function of chloroplasts leading to the chronic photoinhibition [45], i.e., photoprotective mechanisms are not efficient enough in bean leaves due to the excess of excitation energy [57]. We speculate that WB infection, with its increase in the severity scale scores, interferes with chloroplast protein synthesis, as well as with the number of functional reaction centers of PSII, resulting in an increased inactivation of the reaction centers through damaging the processes and/or increasing the rate constant of the non-radiative dissipation of excitation energy [24,27].

### 3.2. Response of the Photosynthetic Apparatus When the Severity Level of Web Blight in Leaves Increases

Among the different responses to disease stress, the adaptive response of the photosynthetic apparatus is important to achieve tolerance to disease [58]. Since photosynthesis provides the plant with energy that is necessary not only for growth, but also for tolerating disease stress, the regulation of photosynthesis should be integrated into the plant’s defense responses to disease [59,60]. For example, in our study, we found that from a WB severity scale score of 2, leaves started to make physiological adjustments to cope with the infection. We observed that there was an effect of infection on the Chl*_a_* fluorescence parameters evaluated (Y(II), Y(NPQ), Y(NO), ETR, qP, qN). Specifically, by increasing the incidence of WB, the quantum yield of the PSII photosystem [Y(II)] was reduced due to the saturation of the reaction centers with an increase in PAR. The decrease in Y(II) was accompanied by a reduction in the electron transport rate (ETR) and the photochemical quenching coefficient (qP), as well as an increase in regulated energy dissipation Y(NPQ) [60]. These photosynthetic adjustments in performance were more pronounced with an increase in PAR as the severity of WB increased. Thus, the interaction of these two factors (disease severity and increasing PAR) also generated an alteration in light saturation dynamics. On the one hand, the effect of infection caused PSII reaction centers to be unable to maintain normal electron transport [61]. Thus, as the disease severity increases, it triggers oxidative damage due to a lack of and/or decrease in energy transfer, which is absorbed by photosynthetic pigments for photochemistry [62]. On the other hand, a higher level of PAR causes photodamage in the plant, which, having a decreased photosynthetic activity due to the infection, enters into a chronic photoinhibition (overload of the protection mechanisms) state that is detected by marked decrease in values of F_v_/F_m_ [63], a situation that was observed for disease severity scale scores of 8 and 9, where photosynthetic activity in the disease affected areas was almost null. When observing the behavior of Y(NPQ), we found certain adjustments due to the incidence of infection. As expected, with an increase in WB severity, there was a greater allocation to regulated energy dissipation Y(NPQ) in the affected areas. This heat dissipation is known to be triggered by the acidification of the chloroplast lumen and the subsequent activation of pH-sensitive enzymes [61], resulting in the conversion of the xanthophyll pigment violaxanthin to antheraxanthin and zeaxanthin, generating changes in the conformation of the light-harvesting complexes surrounding the reaction centers [64,65]. 

The dynamics of Y(NPQ) observed in the disease-affected areas is due to plant pathogenesis impacting the functioning of the chlorophyll-protein complex [66]. As the disease progressively increases in the leaf tissue, the increase in Y(NPQ) triggers a decrease in thylakoid activity [60]. This, in turn, generates a situation that increases the thylakoid proton gradient that modulates the Y(NPQ) process [67,68]. Furthermore, the increase in Y(NPQ) was accompanied by an increase in the qN coefficient from a severity scale score a 3 onwards, reflecting a greater proportion of the absorbed light energy that is dissipated as heat, also indicating a lower efficiency of PSII in using excitation energy for the photochemical reaction [45,69]. However, we found that certain infected leaf regions showed apparent photosynthetic activity, as represented by an increase in Y(NPQ). This could be considered as a defense response of the plant to delay the process of photoinhibition until the severity of disease reaches scale scores of 8 and 9 [68,70]. We found that the stress response was more drastic at severity scores of 8 and 9. We were able to corroborate this by observing a reduction in the qP coefficient, suggesting physical damage to the light-harvesting complex II (LHCII, PSII reaction centers), starting at the PSII reaction center site that is associated with PSII electron transport (inhibiting PSII repair), moving to the water splitting site in the oxygen evolution complex (OEC), or at both sites at the same time [71,72]. In the case of unregulated energy Y(NO), two trends were observed; on the one hand, as PAR increased, Y(NO) decreased, and on the other hand, as the severity level of WB increased, Y(NO) also increased. This situation reduces the photosynthetic efficiency of the plant, without allowing it to improve regulated energy dissipation, inducing physical damage to the chlorophyll–protein complex, causing chronic photoinhibition, and this is because the photoprotective mechanisms are not efficient enough due to excess excitation energy [45].

### 3.3. Relationships between Photosynthetically Active Radiation (PAR) and Photosynthetic Variables at Different Severity Scale Scores of Web Blight

The specific adaptation level of a bean genotype depends on its physiological responses and its degree of susceptibility to a given stress condition [30]. In our study, tropical climatic conditions (higher values of air humidity, rainfall, and air temperature) in the Amazon region are favorable for the frequent development of WB [73,74]. During the initial stages of infection (scale scores of 1 and 2), the elite bean line (BFS 10) faced the combined stress situation (pathogen infection and higher values of PAR) that increases the dissipation of regulated energy and induces a significant reduction in PSII efficiency, while keeping the ETR intact, thus entering into a dynamic state of photoinhibition [63,75]. For severity scale scores of 3 to 7, the PSII Y(II) yield was reduced proportionally with the increase in PAR level from 100 μmol m^−2^ s^−1^, indicating no photosynthetic activity at a PAR of 500 μmol m^−2^ s^−1^. At this point, this behavior would be expected due to the level of damage caused by the disease [60] and due to the inhibition of light-dependent reactions (Y(II), qP and ETR) [76]. For severity scores of 8 and 9, changes in photosynthetic activity were inversely proportional to the severity score of 1. Under these severe disease conditions, the spread of infection interferes with chloroplast protein synthesis, reaching up to a total loss of photosynthetic activity in the leaf regions that were markedly affected by WB stress [77,78].

## 4. Materials and Methods

### 4.1. Experimental Site and Meteorological Conditions

**Figure 8:**
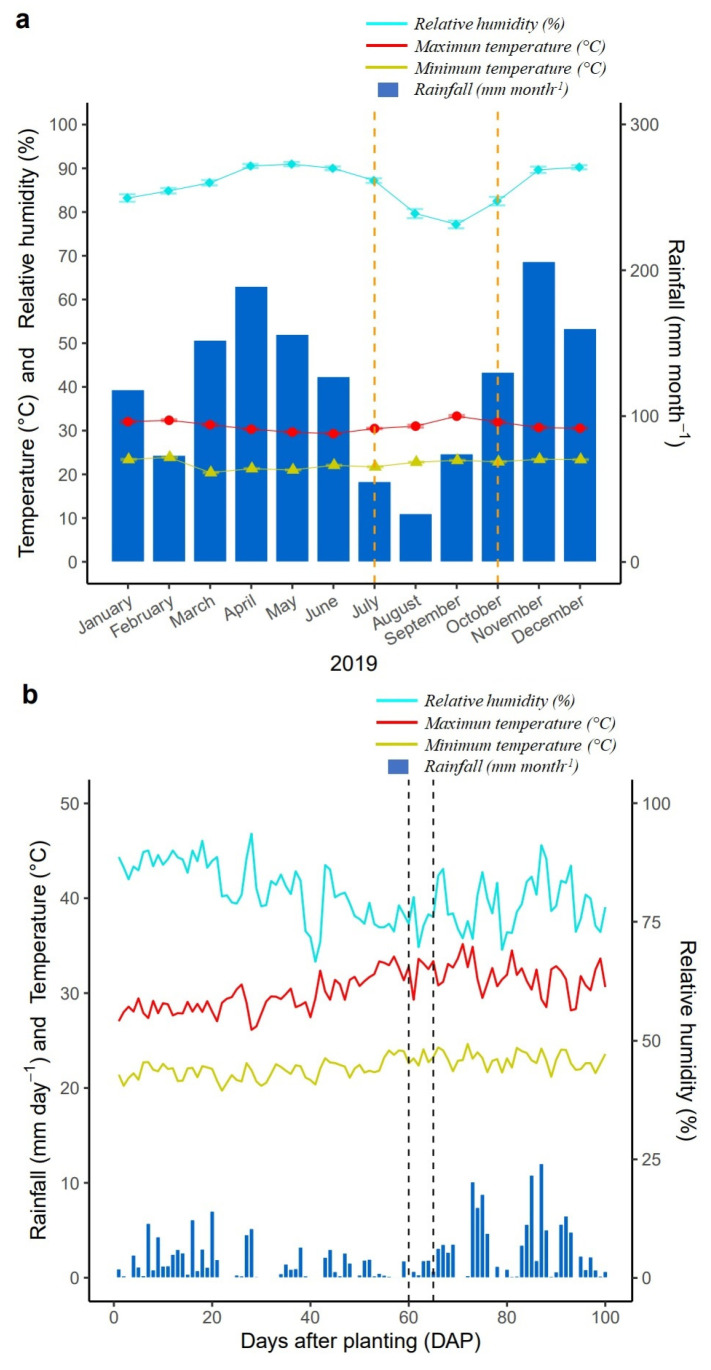
Distribution of rainfall, relative humidity, and maximum/minimum temperatures during: (**a**) the year 2019; (**b**) the crop growth period (July to October) at the CIMAZ Macagual Amazon Research Center in Colombia. The orange dashed vertical lines shown in panel (**a**) indicate the crop growth period (between the months of July and October), and the black dashed vertical lines shown in panel (**b**) indicate the crop growth phase (BBCH 79: between 60 and 65 DAP) during which the chlorophyll fluorescence measurements were obtained.

The experiment was conducted under field conditions during July to October 2019 at the Centro de Investigaciones Amazónicas CIMAZ Macagual of the Universidad de la Amazonia, Colombia (1°37′ N and 75°36′ W). The Center is located in the municipality of Florencia, Caquetá, Colombia, in a tropical rainforest ecosystem. The average annual rainfall is 3800 mm, with maximum rainfall periods in the months of April and November, with an average temperature of 25.5 °C and a relative humidity of 84% with 1700 h of sunshine per year (Figure 8a). During the crop growth period, there was a total precipitation of 196 mm, an average humidity of 80.7%, and an average ambient temperature of 36.4 °C, with minimum temperatures ranging between 19.7–24.6 °C, and maximum temperatures between 26.1–35.1 °C (Figure 8b). This crop growing period is normally used by farmers to avoid excess rainfall and very high humid conditions that favor rapid development of symptoms of WB stress.

### 4.2. Plant Material and Experimental Design

An advanced Mesoamerican common bean breeding line BFS 10 (small red) was used, which has shown good phenological, physiological, and agronomic performance under acidic soil and high temperature stress conditions in the Colombian Amazon [79,80,81,82], and it is also characterized by its adaptation to drought and soils with low fertility. For the present study, a completely randomized plot design was used, with three replications; each replication consisted of 15 rows, each row measuring eight meters long, and the rows were created manually. A row-to-row distance of 0.60 m, with a plant-to-plant spacing of 0.15 m, was used, which was equivalent to 11 plants m^−2^, for a total of 2400 plants in the experimental area, with an experimental area of 216 m^2^. During the crop growing season, weed management was only done manually at 15, 30, and 50 days after planting the crop. No other agronomic management was carried out during crop development. 

### 4.3. Visual Assessment of the Degree of Incidence and Severity of Web Blight 

**Figure 9:**
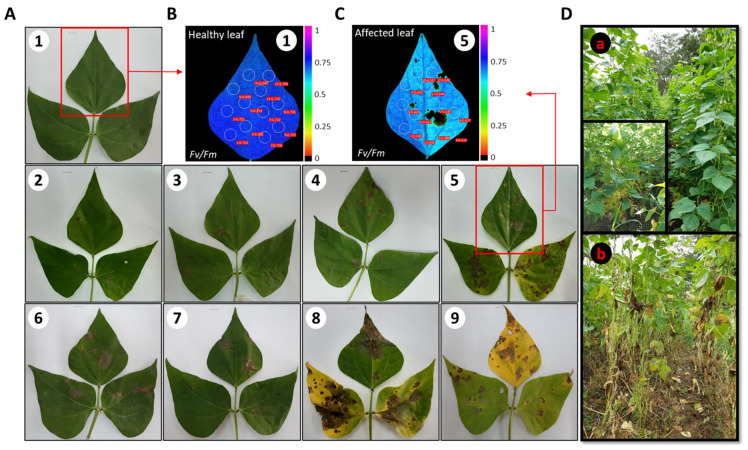
Selection of leaves, leaflets, and locations of areas of interest (AOI) for physiological evaluation in beans: (**A**) different WB severity scale scores (from 1 to 9 according to their degree of visual effect, from 1, no visible symptoms, to 9, as maximum WB severity level; (**B**,**C**) areas of interest (AOI) selected in each leaflet of healthy and infected leaves of *Phaseolus vulgaris*, respectively; figures showing the maximum quantum efficiency of PSII (F_v_/F_m_) in healthy and infected leaves, respectively; the variations in the color gradient from violet to red (from 1 to 0) indicate the decrease in value of the F_v_/F_m_ ratio as a response to disease stress; (**D**) (**a**) State of disease development for the leaf samples taken following the BBCH scale for beans, which corresponds to the BBCH 79 crop growth stage [84] with a close-up view of disease development; and (**b**) advanced stage of disease stress, with severe disease symptoms (scale 9).

Using the standard scale system for the evaluation of bean germplasm proposed by Schoonhoven and Pastor-Corrales [11], the incidence and severity of web blight (WB) were evaluated visually (Figure 9A; visual scale score ranging from 1 to 9). The incidence was determined from the number of plants per plot (considered as each replicate consisting of 15 rows) with signs of disease over the total number of plants per plot (n = 475 plants in total) per 100. The severity was determined by the plants with signs of disease in each plot, considering the degree of the affected region on the leaf, with 1 (0%) as the level of severity in which there is no visual presence of WB on the leaf lamina up to 9 (100%) as the maximum level of disease incidence (Figure 9A [11]). The severity was measured during the period of mid-pod filling (individual seeds visible in the first pods, BBCH 79, between 60–65 DAP, Figure 9D(a)), when WB is most rapidly spread [83] (Figure 9D(a)) and during which the deterioration caused by the pathogen at the physiological level can be evaluated. For this purpose, 9 leaves with visual presence of WB (one for each severity scale score from 1 to 9) were selected per plant in each plot (n = 9 plants per plot) (Figure 9A). Leaves without a visual presence of WB corresponding to scale score 1 (0% WB affectation) were taken between the fifth and eighth trifoliate leaf (from apex to base) (Figure 9A(1)), and leaves affected by the pathogen (from scale 2 to 9) (Figure 9A(2–9)) were selected between the lower and middle stratum in plants where WB development and advancement were evidenced (Figure 9D). Chlorophyll fluorescence measurements were obtained on the central leaflet in each leaf for each of the scales of WB affected leaves (Figure 9B). In each leaflet, 12 circular areas of interest (AOI), distributed in the lower, middle, and upper part of the leaflet, were observed, and the AOI in the affected leaves were located next to the lesions, but not on them (Figure 9C); thus, the areas in which the information of the different chlorophyll fluorescence variables was obtained. In total, 2916 data were obtained for each variable, corresponding to 12 AOI in 243 leaves (9 leaves × 9 plants × 3 replicates).

### 4.4. Determination of Chlorophyll Fluorescence (Chl_a_) Parameters in Leaves Affected by Web Blight

The pathogen stress incidence was monitored using a MAXI-Imaging PAM M-Series fluorometer (Heinz Walz GmbH, Effeltrich, Germany), equipped with 44 high-power royal blue (450 nm) LED lamps, which provide modulated excitation light, as well as actinic illumination and saturation pulses. Chlorophyll *a* (Chl*_a_*) fluorescence emissions were captured with a 640 × 480 pixel resolution CCD (charge-coupled device) camera with a visible sample area of 24 × 32 mm on each leaf. From the color gradients and trends obtained with increasing photosynthetically active radiation (PAR) captured using Imaging WIN version 2.32 software (Heinz Walz GmbH, Effeltrich, Germany), different parameters related to chlorophyll fluorescence (Chl*_a_*) were determined. The bean leaves with signs of WB infection selected for the fluorescence measurement were detached from the plant between 07:00 and 08:00 am, when the plants did not present saturating light incidence or stress conditions due to high temperatures. Subsequently, the leaves were subjected to a 60 min dark adaptation process in order to completely open the PSII reaction center and minimize the dissipation of non-photochemical energy. After the dark adaptation process was completed, the leaves were individually fixed at a distance of 18.5 cm from the chamber, where they were exposed to a weak, modulated light beam (0.5 μmol m^−2^ s^−1^, 100 μs, 1 Hz) in order to determine the initial fluorescence (F_0_), and a saturating pulse of white light was immediately emitted (2400 μmol m^−2^ s^−1^, 10 Hz) for 0.8 s to determine the maximum fluorescence emission (F_m_), or when all PSII reaction centers are expected to be “closed.” Next, the steady-state chlorophyll fluorescence yield (F_s_) of the plants under actinic illumination was determined and a second saturating pulse was applied, with the same settings used for determining F_m_, to measure their maximum light-adapted fluorescence yield (F_m_′). 

Using the above protocol, different Chl*_a_* parameters were determined in dark-adapted and light-adapted states. First, the maximum quantum yield of PSII (F_v_/F_m_ = (F_m_ − F_0_)/F_m_) was calculated. Likewise, the energy pathways in yield fractions corresponding to the following were calculated: i. effective quantum yield of PSII (Y(II) = (F_m_′ − F_0_)/F_m_′) [85]; ii. quantum yield of regulated energy dissipation in the form of non-photochemical heat of PSII (Y(NPQ) = 1 − Y(II) − 1/(NPQ + 1 + qL (F_m_/F_0_ − 1)) [41]; and iii. the quantum yield of non-regulated energy dissipation of PSII (Y(NO) = 1/(NPQ + 1 + qL (F_m_/F_0_ − 1)) [41], where qL is the coefficient of photochemical quenching, and NPQ is the non-photochemical quenching. From the same protocol, the level of fluorescence emissions of PSII was determined using rapid light curves (RLC) in 13 steps, with a duration of 20 s at each irradiance level (0, 1, 21, 56, 111, 186, 281, 336, 396, 461, 531, 611, 701 µmol photons m^−2^ s^−1^). At each point of the curve, parameters such as the electron transport rate (ETR), acting as an indicator of the photosynthetic capacity of live plants, were calculated (ETR = 0.5 × Y(II) × PAR × 0.84 µequivalents m^−2^ s^−1^) where: “0.5” refers to the stoichiometric distribution of the absorbed light energy between the two photosystems, “Y(II)” to the effective quantum yield of PSII, and “PAR” to the incident photon flux density µE m^−2^ s^−1^, and “0.84” is considered as the empirical absorption efficiency of 84% of the incident light in plants [85]. The photochemical quenching coefficient ((qP = (F_m_′ − F)/(F_m_′ − F_0_′)), where F_0_″ is the minimum fluorescence yield in light F_0_′ = F_0_/(F_v_/F_m_ + F_0_/F_m_′)) [86], which is the amount of energy used in the photochemical pathway, was also calculated [86], as well as the non-photochemical quenching coefficient (qN = (F_m_ − F_m_′)/(F_m_ − F_0_′) [86]), which provides an indication of early the detection of stress-induced limitations.

### 4.5. Quantification of the Incidence of Web Blight on the Functioning of the Photosynthetic Apparatus

From the F_v_/F_m_ images obtained with the MAXI-Imaging PAM M-Series fluorometer (Heinz Walz GmbH, Effeltrich, Germany) and using the Imaging WIN software version 2.32 (Heinz Walz GmbH, Effeltrich, Germany), we proceeded to calculate: (i) the visually affected leaf area (%), and (ii) the area of the leaf lamina (%) where PSII is not functioning, the latter allowing for the effective identification of the magnitude of the incidence or damage of the pathogen on the photosynthetic apparatus at each level of WB severity. Using the analysis function provided by ImagingWIN, the analysis scale was created by delimiting the range between 0.0 and 1.0 (low to high), which proceeds from a gradient of black to red, allowing this contrast to determine the area, both in pixels and in mm^2^. For this purpose, the relationship between the area in pixels and mm^2^ was analyzed, and a linear function was obtained showing the area of the leaf in mm^2^ (Y) as a variable dependent on the number of pixels in the image (Y = 0.0423X + 3 × 10^−12^, r^2^ = 0.999, *p* < 0.001). For the calculation of the area affected by the pathogen in relation to the functioning of the photosynthetic apparatus, Y(II) was used, taking 0.157 as the minimum contrast value, which allowed for obtaining the area and the number of pixels for each PAR level (0, 1, 21, 56, 111, 186, 281, 336, 396, 461, 531, 611, 701 µmol photons m^−2^ s^−1^). Both for obtaining the visibly affected area and for determining the area of the affected photosynthetic apparatus, the images were analyzed in ImageJ software (1.52u, National Institutes of Health, NIH, Bethesda, MD, USA), following the method proposed by various authors [24,87,88], where the area without visual signs of disease was calculated, as well as the necrotic areas generated by WB. A total of 243 F_v_/F_m_ images of the same number of leaves (9 leaves from 9 plants with 3 replicates, i.e., 27 leaves at each WB severity scale) were used. In addition to the determination of the area or region affected by WB, transects of 160 pixels wide were made, in which F_v_/F_m_ were compared in both healthy and affected areas, allowing for quantitatively observing the impact of the incidence of the pathogen on the function of the photosynthetic apparatus. 

### 4.6. Statistical Analysis

To evaluate the differences in F_v_/F_m_, Y(II), Y(NPQ), Y(NO), ETR, qP, and qN between the different severity scales of WB, a linear mixed model (LMM) was fitted. For this purpose, the fixed effects within the LMM were severity level and PAR, as well as their interaction. The AOI, leaves, and plants within the plots were considered as random effects. The assumptions of normality and homogeneity of variances were validated by means of an exploratory analysis of the residuals. Likewise, a Fisher’s LSD post-hoc mean comparison test was performed, with a significance of α = 0.05. The graphs were displayed in quadrants with the corresponding means and statistical significance on each axis. To determine the relationship between the disease severity scale and the photosynthetic variables, a Pearson’s correlation analysis was performed, and the results were presented graphically using a heat map at the general level, and a chord diagram was used for the most contrasting severity scales using the “corrplot” [89] and “circlize” [90] packages. LMMs were performed with the lme function of the nlme package, and graphical outputs were performed in the packages “ade4”, “ggplot2”, “factoextra”, and “dplyr” in R language software, version 3.4.4 (R Foundation for Statistical Computing, Vienna, Austria) [91], using the interface in InfoStat [92].

## 5. Conclusions

In this study, we evaluated the fluorescence response of the photosynthetic apparatus to increased disease severity caused by web blight (*Thanatephorus cucumeris* (Frank) Donk) on common bean (*Phaseolus vulgaris* L.) leaves under acidic soil and the humid tropical conditions of the Colombian Amazon. We found a significant effect of web blight (WB) on the functional status of the photosynthetic apparatus. The marked influence on photosynthesis was apparent from a mild disease severity scale score of 2, where the use of energy devoted to the photosynthetic machinery was reduced by up to 50%, in comparison with a scale score of 1. The results from this study indicated that the use of fluorescence imaging could serve as an effective method to reliably quantify the impact of the incidence of WB on the functional status of the photosynthetic apparatus in the common bean. However, further research is needed for evaluating a breeding population developed for WB resistance under controlled and natural conditions of disease development. This effort could increase the value of this physiological phenotyping as a screening tool for bean breeders aimed at the early detection of disease resistance in breeding lines.

## Data Availability

Data are available from the authors upon request.

## Supplementary Materials

The following supporting information can be downloaded at: https://www.mdpi.com/article/10.3390/plants11233238/s1, Figure S1. Rapid light curves (RLC) for fluorescence parameters, including (a). effective quantum yield in photosystem II Y(II), (b). quantum yield of regulated energy dissipation Y(NPQ), (c). quantum yield of unregulated energy dissipation Y(NO), (d). apparent electron transporting rate (ETR), (e). coefficient of photochemical quenching (qP), and (f). coefficient of non-photochemical quenching (qN), to evidence the physiological deterioration caused by web blight in bean leaf blades with an increase in its degree of severity and an increase in photosynthetically active radiation (PAR). Results include mean ± SE (n = 144). Table S1. Mean values of chlorophyll *a* fluorescence parameters. For the variables of: (F_0_)—initial fluorescence; (F_m_)—maximum fluorescence; (F_v_/F_m_)—maximum quantum yield of PSII; Y(II)—effective quantum yield of PSII photosystem; Y(NPQ)—quantum yield of regulated energy dissipation in the form of non-photochemical heat of PSII; Y(NO)—quantum yield of non-photochemical energy dissipation of PSII; (ETR)—electron transport rate; (qP)—photochemical quenching coefficient; and (qN)—non-photochemical quenching coefficient, in response to the increasing degree of the effect of web blight fungus (severity scales) and the increasing photosynthetically active radiation (PAR) through rapid light curves (RLC).
