# Supplementary material for: Impact of Web Blight on Photosynthetic Performance of an Elite Common Bean Line in the Western Amazon Region of Colombia
Source: Plants (Basel). 2022 Nov 25;11(23):3238. doi: 10.3390/plants11233238 (PMC9736428; doi:10.3390/plants11233238)
Supplement: Supplementary file 1 [file plants-11-03238-s001.zip › Supplementary Table 1.docx]

**Supplementary Table 1.** Mean values of chlorophyll a fluorescence parameters. For the variables of: (F_0_). initial fluorescence; (F_m_). maximum fluorescence; (F_v_/F_m_). maximum quantum yield of PSII; Y(II). effective quantum yield of PSII photosystem; Y(NPQ). quantum yield of regulated energy dissipation in the form of non-photochemical heat of PSII; Y(NO). quantum yield of non-photochemical energy dissipation of PSII; (ETR). electron transport rate; (qP). photochemical quenching coefficient; and (qN). non-photochemical quenching coefficient; in response to increasing degree of web blight fungus effect (severity scales) and increasing photosynthetically active radiation (PAR) through rapid light curves (RLC).


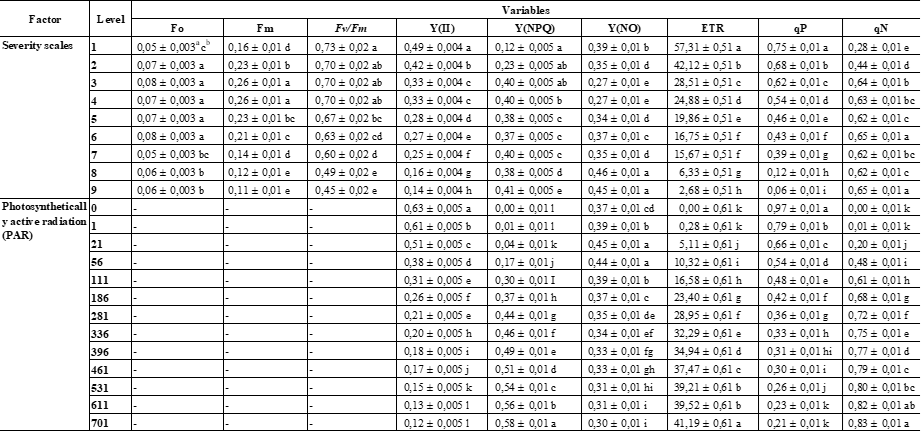


Means in each column followed by the same letter not differ statistically (Fisher's LSD test, p<0.005)
