# Supplementary figures and images for: Impact of Web Blight on Photosynthetic Performance of an Elite Common Bean Line in the Western Amazon Region of Colombia
Source: Plants (Basel). 2022 Nov 25;11(23):3238. doi: 10.3390/plants11233238 (PMC9736428; doi:10.3390/plants11233238)

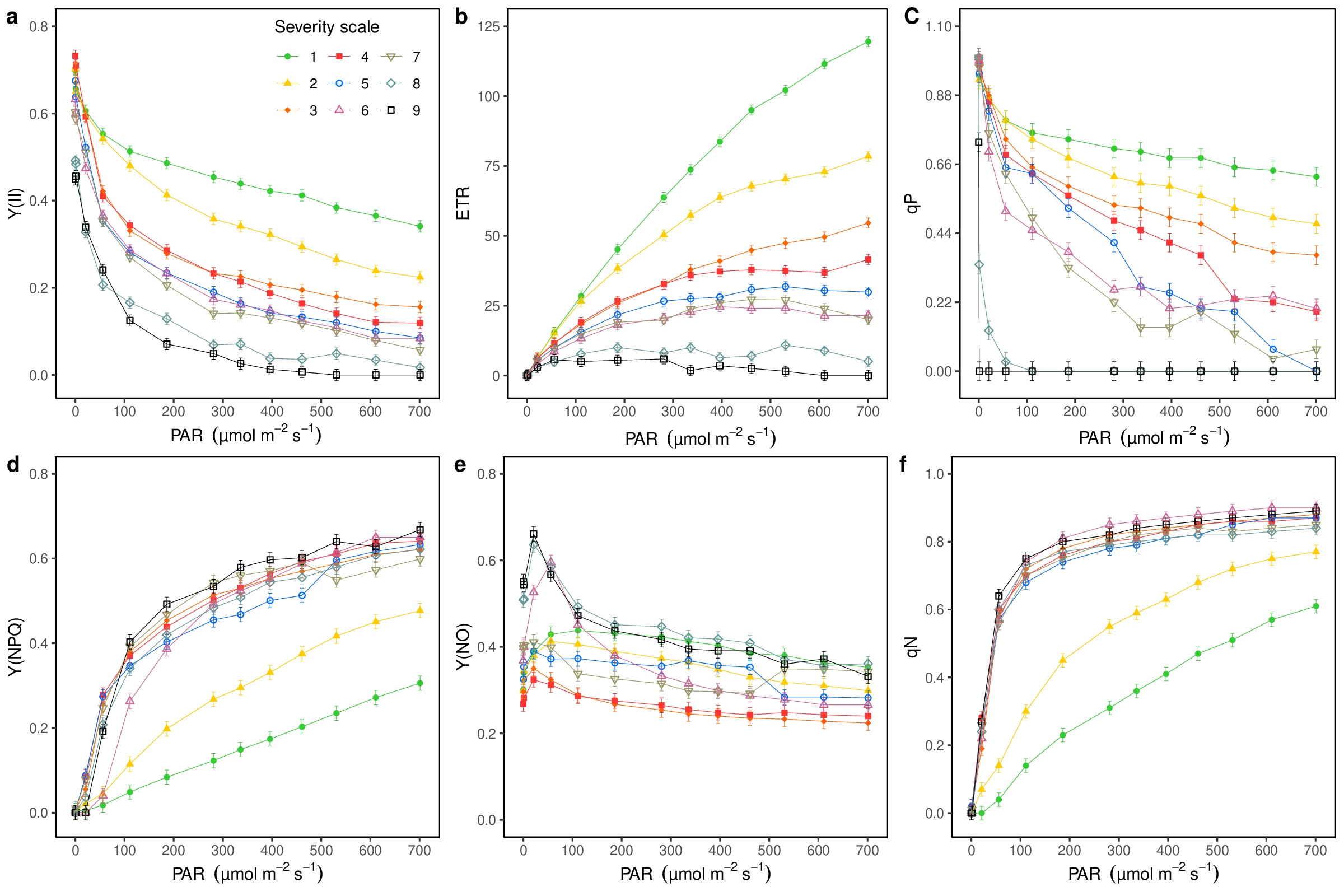

Supplement: Supplementary file 1 [file plants-11-03238-s001.zip › Figure S1.jpg]
